# Atypical Pressure Dependent Structural Phonon and Thermodynamic Characteristics of Zinc Blende BeO

**DOI:** 10.3390/ma18153671

**Published:** 2025-08-05

**Authors:** Devki N. Talwar, Piotr Becla

**Affiliations:** 1 Department of Physics, University of North Florida, 1 UNF Drive, Jacksonville, FL 32224, USA; 2 Department of Physics, Indiana University of Pennsylvania, 975 Oakland Avenue, 56 Weyandt Hall, Indiana, PA 15705, USA; 3 Department of Materials Science and Engineering, Massachusetts Institute of Technology, Cambridge, MA 02139, USA; becla@mit.edu

**Keywords:** novel meta stable BeO, rigid-ion model, lattice dynamics at ambient and high pressure, thermodynamical properties, Raman scattering, infrared spectroscopy

## Abstract

Under normal conditions, the novel zinc blende beryllium oxide (zb BeO) exhibits in a metastable crystalline phase, which is less stable than its wurtzite counterpart. Ultrathin zb BeO epifilms have recently gained significant interest to create a wide range of advanced high-resolution, high-frequency, flexible, transparent, nano-electronic and nanophotonic modules. BeO-based ultraviolet photodetectors and biosensors are playing important roles in providing safety and efficiency to nuclear reactors for their optimum operations. In thermal management, BeO epifilms have also been used for many high-tech devices including medical equipment. Phonon characteristics of zb BeO at ambient and high-pressure P ≠ 0 GPa are required in the development of electronics that demand enhanced heat dissipation for improving heat sink performance to lower the operating temperature. Here, we have reported methodical simulations to comprehend P-dependent structural, phonon and thermodynamical properties by using a realistic rigid-ion model (RIM). Unlike zb ZnO, the study of the Grüneisen parameter γ(T) and thermal expansion coefficient α(T) in zb BeO has revealed atypical behavior. Possible reasons for such peculiar trends are attributed to the combined effect of the short bond length and strong localization of electron charge close to the small core size Be atom in BeO. Results of RIM calculations are compared/contrasted against the limited experimental and first-principle data.

## 1. Introduction

Novel alkaline earth II-oxides (XO: X = Be, Mg, Zn and Cd) occur in different crystalline structures ranging from the wurtzite (wz) → zinc blende (zb) → rock salt (rs) phases. These II-O semiconductors are considered valuable in a broad range of applications [1,2,3,4,5,6]. Unlike other XOs, the beryllium oxide (BeO), commonly known as “beryllia”, has attracted significant attention in recent years due to its fascinating electrical, mechanical and thermal properties. BeO possesses a relatively low density, largest wide bandgap (WBG) $E_{g}\;\sim 10.6\;eV,$ high rigidity, high melting point 2570 °C and strong chemical bond. As a ceramic, it exhibits an electrical insulating feature with exceptional thermal conductivity κ, and high temperature stability [7,8,9,10,11]. Unique basic characteristics of BeO offered major contributions to materials science, condensed matter physics and electrical engineering. These traits of BeO encouraged many physicists and chemists to investigate its electronic, elastic, thermal and phonon features at ambient and high pressure [1,2,3,4,5,6]. Many BeO-based electronic components are developed such as ultraviolet (UV) detectors, light emitting diodes (LEDs), laser diodes (LDs), gas sensors and solar cells, etc. [1,2,3,4,5,6,7,8,9,10,11] It has contributed to lasers for DNA sequencing and tissue analysis. As a UV transparent conducting oxide (TCO), BeO has also been employed in flat-panel displays. Recently, the BeO-based devices are methodically incorporated (i) in satellites/aircraft for space exploration, (ii) in nuclear reactors as a neutron reflector, (iii) in medical equipment for radiation detection and (iv) for managing excess heat generation during the operations of electronic and optoelectronic systems [7,8,9,10,11].

In BeO, the prospects of spontaneous polarization by an external electric field E can alter intrinsic defect structures [7,8,9,10,11]. Although not enough conclusive evidence exists for spontaneous polarization modifying impurity configurations, it is theoretically possible from the available research on related materials. An interplay between E and intrinsic defects is a complex subject and requires further study. Nevertheless, it has been substantiated that a change of defect structure can alter bulk and surface characteristics of BeO. These changes are known to radically impact on the performance of electronic devices [7,8,9,10,11]. As a gate dielectric and oxygen diffusion barrier, an extremely assuring high-k dielectric of BeO has been used for designing metal–oxide–semiconductor field effect transistors (MOSFETs) [1,2,3,4,5,6]. As a well-known refractory material, BeO is employed as a heat sink and substrate in many high-power electronic devices to effectively dissipate heat and prevent overheating [12,13,14,15]. In nuclear reactors, BeO has served as a dispersion phase fuel matrix (DPFM) due to its good compatibility with uranium dioxide UO_2_. The DPFM has not only improved the overall performance of reactors but also provided safety to its fuel. In band gap engineering, BeO is frequently mixed with other iso-structural XOs to prepare ternary X_x_Be_1−x_O and quaternary X_x_Y_y_Be_1−x−y_O alloys. In low-dimensional heterostructures (LDHs) [viz., multi quantum wells (MQWs) and superlattices (SLs)], the integration of BeO-based epifilms has offered unlimited opportunities to develop electro-optical device structures. Recently, many photonic devices have been meticulously blended for improving the flexible micro-/nano-electronics [1,2,3,4,5,6].

To prepare ultrathin BeO epifilms, different growth methods have been employed [16,17,18,19,20,21,22,23,24,25,26,27,28]. Commonly used epitaxial techniques include the chemical vapor deposition (CVD), metal organic (MOCVD) [16,17], laser ablation [18,19,20], molecular beam epitaxy (MBE), pulsed laser deposition (PLD), atomic layer deposition (ALD), plasma-enhanced ALD (PEALD), plasma-assisted PA-MBE [21,22,23,24,25,26,27,28], etc. During BeO growth, achieving precise control over its stoichiometry and maintaining high crystalline quality can be challenging as it requires high oxidation potentials and high crystallization temperatures T. Choosing an appropriate substrate is equally crucial. Ideally, the substrate requires having a compatible crystal structure and lattice parameters to minimize strain for promoting high-quality growth. Nonpolar and low-symmetry surfaces are preferred in the MBE growth to avoid twinning. Volatility of Be at the film surface in the PLD method requiring high growth T, limit maximum achievable T could impact BeO film growth rate and quality. Despite recent achievements of preparing epifilms, a few issues still persist: As a toxic element, Be requires strict safety precautions and specialized handling procedures for the BeO growth due to health hazard issues. Being a rare element, Be can cause continuous supply challenges with possible price volatility of the BeO-based devices.

Systematic characterization efforts have recently been made [29,30,31,32,33,34,35,36,37,38,39,40,41,42,43,44,45,46,47,48,49,50,51] for comprehending the basic traits of BeO epifilms. An in-situ reflection high-energy electron diffraction (RHEED) technique in MBE growth of BeO thin film allowed a real-time evaluation of its morphology and surface structure [29,30]. In epifilms, Auger electron spectroscopy (AES) [31] is considered for analyzing its surface composition. He^+^-Rutherford backscattering spectrometry (RBS) [32] helped to determine the distribution of impurity and/or contamination atoms at different depths within a few micrometers from the BeO film surface. For advanced semiconductor-based electronic device applications, an atomic force microscopy (AFM) is used for characterizing various aspects of epifilms [33,34]. High-resolution X-ray diffraction [34,35] (HR-XRD) helped to differentiate between amorphous and crystalline forms of BeO. Cross-sectional transmission electron microscopy (XTEM) is employed, analyzing BeO thin films at a very fine scale. In photoluminescence (PL), the absorption and emission of light in solids at a longer wavelength provides energy bandgap $E_{g}$ [36,37,38,39,40]. Fourier-transform infrared (FTIR) [41,42] and Raman scattering spectroscopy (RSS) [43,44,45,46,47,48] offer phonon behavior of BeO under varying conditions including high T and pressure P. Spectroscopic ellipsometry (SE) in BeO epifilms has been employed for assessing its thickness, composition and optical properties [49,50,51].

Despite the successful growth [16,17,18,19,20,21,22,23,24,25,26,27,28] and characterization [29,30,31,32,33,34,35,36,37,38,39,40,41,42,43,44,45,46,47,48,49,50,51] of epifilms, there are still many basic issues of BeO-based device structures that remained unresolved. For instance, the structural, electronic, elastic and vibrational traits of epitaxially grown films have not yet been thoroughly investigated. The study of lattice dynamics of BeO is important for understanding its role in different device performance. Vibrational properties are crucial for evaluating electron–phonon interactions, transport coefficients, as well as assessing the reliability and functionality of electronic devices. Among other XOs, the phonons in BeO are the major heat carriers [7,8,9,10,11]. In designing device structures, the zb phase is preferred due to its unique optical and electronic properties. Specifically, the zb BeO offers many advantages of lower carrier scattering and higher doping efficiencies as compared to the wz structure. The zb BeO is known to have a higher κ value for causing higher thermal properties as compared to the wz BeO [7,8,9,10,11].

In solids, an inelastic neutron scattering (INS) spectroscopy is considered as one of the most powerful experimental techniques to study the frequency–wavevector relationships of phonons [47]. Careful analysis of energy lost and/or gained by scattered neutrons in INS helps in determining the phonon dispersions, $\omega_{j}\left(\vec{q}\right)$. Except for zb ZnO and wz ZnO [47], no experimental results exist for the lattice dynamics of zb BeO. In the absence of INS measurements, RSS and FTIR spectroscopies are commonly employed [41,42,43,44,45,46,47,48] for assessing phonon frequencies at high critical points in the Brillouin zone (BZ). In crystals with free charge carriers, Raman scattering has also been exploited for comprehending carrier–phonon interactions and phonon-assisted optical transitions [47,48,49,50,51]. In polar materials, while RSS perceives inelastic scattering of light by phonons, FTIR spectroscopy measures the absorption of infrared light by the vibrational modes.

High-pressure studies with diamond anvil cells (DACs) have proven valuable for investigating the structural and vibrational properties of different materials. Both RSS and FTIR spectroscopies are employed for comprehending P-dependent shifts of phonons. Analyses of such results have helped gain valuable insights of the bonding characteristics of materials (i.e., hardening and/or softening of inter-molecular/intra-molecular interactions) including their phase transitions $P_{t}$ Raman scattering spectroscopy results are also exploited to understand the T-dependent mode Grüneisen parameter$\;\gamma \left(T\right).$ The information of $\gamma \left(T\right)$ in solids at a given T signifies the sensitivity of P-dependent phonons to its volume change. The P- and/or T-dependent vibrational studies can help understand the optical and thermodynamical properties of materials including specific heat $C_{v}(T)$, Debye temperature $\theta_{D}\left(T\right),$ thermal expansion coefficient (TEC) α(T), etc. [6].

From theoretical standpoints, several ab initio calculations are performed for the wz and rs BeO [52,53,54,55,56,57,58,59,60,61,62,63,64,65,66]. Typically, first-principle simulations have employed either a density functional theory (DFT) and/or the density functional perturbation (DFP) methods. Calculations are performed using full potential linear augmented plane wave (FP-LAPW), frozen phonon (FP), molecular dynamics (MD) and ab initio (AI-MD) schemes [52,53,54,55]. Very few studies are available for BeO in the zb phase. Some of the reported results on zb ZnO for electronic and vibrational characteristics are either inconsistent and/or questionable [52,53]. For instance, Chibueze [52] has recently adopted an ab initio approach to study the mechanical, phonon and electronic properties of zb ZnO. In the framework of DFT, the author [52] employed a generalized gradient approximation (GGA) by considering a revised Perdew–Burke–Ernzerhof GGA method. For zb ZnO, [52] it is claimed that degenerate phonon energies at the center of the BZ [i.e., near $\vec{q}\to 0,\omega_{LO\left(\Gamma \right)}=\omega_{TO\left(\Gamma \right)}\sim 379{cm}^{-1}]$ is achieved. This result is in complete disagreement with the INS [48,49] as well as the first-principle theoretical results of phonon dispersions $\omega_{j}(\vec{q})$ [49,54]. Absolutely no attempts are made to comprehend the P-dependent $\omega_{j}\left(\vec{q}\right)$ or thermodynamical characteristics of zb BeO. Systematic studies are required to decide whether the material is stable or prone to instability. We anticipate that the accurate results of BeO will play vital roles in evaluating its use in high-T applications, especially for thermal management needs in nuclear reactors [7,8,9,10,11].

This paper aims to report the results of a methodical study on the novel zb BeO material by exploiting a rigid-ion model (RIM) [67,68,69,70,71,72,73]. Calculations are performed to understand the structural, phonon and thermodynamical properties of zb BeO at ambient and high P. In Section 2.1, different crystal structures of BeO are described. The salient feature of RIM is outlined in Section 2.2 and Section 2.2.1, Section 2.2.2 and Section 2.2.3 with complete details reported elsewhere [67]. The model includes both the short-range and long-range Coulomb interactions. Unique optimization procedures [68] are applied to estimate the interatomic force constants (IFCs) at P = 0 GPa and P = 20 GPa [74,75,76,77,78,79]. To achieve an improved set of IFCs for zb BeO, we have incorporated accurate values of phonon frequencies at Γ, X and L critical points in the BZ and P-coefficients ∂$\omega_{j}\left(\vec{q}\right)$/∂P as the input, while employing the elastic constants $c_{ij}$, their pressure derivatives ∂$c_{ij}$/∂P (cf. Section 3, Section 3.1 and Section 3.2), equilibrium lattice-constants $a_{0}$ and P-dependent $a/a_{0}$ as constraints. For zb BeO and using Murnaghan’s equation of state [74], we have simulated the P-dependent volume $V/V_{0}$ and/or lattice constant ratio $a/a_{0}$ by considering appropriate values of bulk modulus $B_{0}$ and its pressure derivative $B_{0}^{\prime }$ (cf. Section 3, Section 3.1 and Section 3.2). In zb BeO, the RIM results of phonon dispersions $\omega_{j}\left(\vec{q}\right)$ and one-phonon g(ω) density of states (DOS) at P = 0 GPa, and P = 20 GPa are systematically employed in the quasi-harmonic approximation (QHA) to study (Section 3, Section 3.1 and Section 3.2) its thermodynamical properties [e.g., $\theta_{D}$(T) and $C_{V}$(T)]. By incorporating the results of Grüneisen dispersions $\gamma_{j}\left(\vec{q}\right)$, we have meticulously achieved the T-dependent Grüneisen parameter γ(T) and linear thermal expansion coefficient α(T). Unlike zb ZnO, the analysis of γ(T) and α(T) for zb BeO has revealed atypical behavior (Section 3, Section 3.1 and Section 3.2). Possible explanation for such peculiar trends is ascribed to the combined effect of the short bond length and strong electron localization due to small core size of the electronic charge close to Be in BeO. The results of RIM calculations are compared/contrasted against the existing experimental and first-principles data [80,81,82,83], with concluding remarks presented in Section 4.

## 2. Theoretical Background

### 2.1. Structural Properties

**Figure 1:**
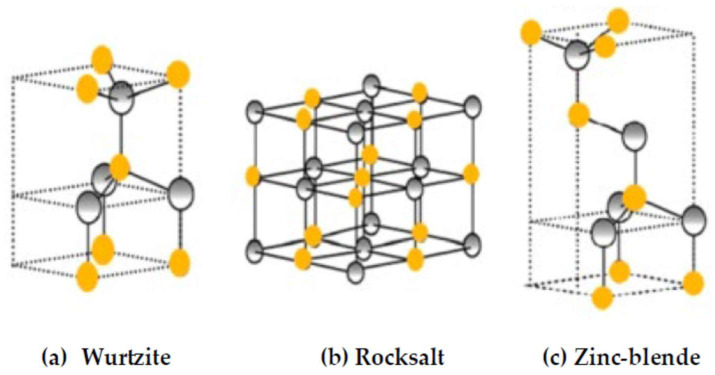
Three possible crystal structures of XOs: (**a**) wurtzite (B4), (**b**) rock salt (B1) and (**c**) zinc blende (B3). The X and O atoms are shown by using shaded grey and solid yellow color spheres, respectively.

Unlike other XOs, the earlier P-dependent measurements for BeO have exhibited two main crystal phases: (i) the hexagonal wz (B4) $P6_{3}mc\left(C_{6v}^{4}\right)\;$and (ii) the sodium chloride or rock salt (rs) (B1) $Fm\bar{3}m\left(O_{h}^{5}\right)\;$structures. For low P and T (<2500 K), while BeO occurs in the most stable wz structure it can attain, the rs phase at higher P and T [1,2,3,4,5,6]. By using an X-ray synchrotron radiation source, Mori et al. [75] carried out phase transition studies under high P. The authors have established that the phase transition $P_{t}$ appears at ~137 GPa from wz B4 → rs B1. Structural changes did not evolve for P ≤ 55 GPa. No phase transition$\;P_{t}$ is detected from wz B4 → zb (B3) $F\bar{4}3m\left(T_{d}^{5}\right)\;$[75]. For theorists, the studies of $P_{t}$ have also faced considerable challenges [84,85,86,87,88,89,90,91,92,93,94]. Unlike experimental studies, the calculations in BeO [84,85,86,87,88,89,90,91,92,93] predicted $P_{t}$ from wz (B4) → zb (B3) → rs (B1). However, the projected values of $P_{t}$ are perceived varying between 22 GPa $<P_{t}$ <147 GPa. In Figure 1, we have displayed the three possible crystal structures of XOs.

#### Phase Transition

Several DFT simulations are available for the BeO material [84,85,86,87,88,89,90,91,92,93] to comprehend its structural phase transitions from B_4_ → B_3_ → B_1_. The studies have predicted significantly different values of $P_{t}$. From these calculations, an average value of $P_{t}\;$has been established at nearly ~84 GPa. A soft nonlocal pseudopotential method in BeO was adopted earlier by Van Camp et al. [92] in the local density function (LDF) approximation. The authors [92] projected the phase transitions $P_{t}\;$from B4 → B3 and B3 → B1 at ~74 and ~137 GPa, respectively. Using an all-electronic and full-potential electronic structure method in the framework of LDF, Boettger et al. [93] reported the corresponding transitions at $P_{t}\;\sim$63–76 GPa and $P_{t}\;\sim$95 GPa. Later, Park et al. [91] suggested the phase transitions in BeO from B4 → B3 at $P_{t}\;\sim$91 GPa, and B3 → B1 at $P_{t}\;\sim$147 GPa by adopting a first-principles soft nonlocal pseudopotential method in the generalized gradient approximation. In Table 1, we have listed the calculated values of the equilibrium $P_{t}\;$obtained by several researchers for the BeO material between B_4_ → B_3_ → B_1_ using different ab initio methods [84,85,86,87,88,89,90,91,92,93]. Obviously, the high-pressure status of BeO has been and still is extremely ambiguous [75,84,85,86,87,88,89,90,91,92,93].

### 2.2. Computational Methodology of Lattice Dynamics

In a crystal lattice, the simulated phonon dispersions $\omega_{j}\left(\vec{q}\right)\;$are used to describe its collective atomic vibrations. In semiconductors, the study of lattice dynamics has played a crucial role for understanding their structural, thermal, optical and electrical properties. For comprehending $\omega_{j}\left(\vec{q}\right)\;$in zb materials, two types of theoretical methods are frequently employed: (i) the microscopic or first-principle approaches [52,53,54,55,56,57,58,59,60,61,62,63,64,65,66,67], and (ii) the macroscopic techniques [68,69,70,71,72,73,74]. Phonon dispersion studies in solids using microscopic theories rely on their fundamental physical principles which allow for a detailed understanding of atomic interactions. Macroscopic schemes have, however, employed phenomenological models which require systematic evaluation of the general interatomic force constants. The later methods often used the simplified representations for numerical evaluation of IFCs by linking them to the material’s fundamental traits rather than examining via individual atomic interactions.

In essence, the microscopic techniques provide substantial details for the vibrational characteristics of materials, while the macroscopic methods offer simplified and efficient perspectives through IFCs to study the lattice dynamical features. In zb ZnO [94,95] and BeO [96], we have exploited a macroscopic RIM (cf. Section 2.2.1) and methodically obtained the optimized sets of its eleven IFCs (cf. Section 2.2.3) at ambient P by incorporating accurate values of phonons (see Table 2), elastic constants $c_{ij}$ and lattice constant $a_{o}.$

#### 2.2.1. Rigid-Ion Model

The macroscopic model that we adopted here to study the vibrational characteristics of zb BeO includes both the short-range (up to second-nearest neighbors) and long-range Coulomb interactions [67]. In RIM, an atom is identified using two indices: *l* and κ. The term *l* represents the number of unit cells while κ signifies the types of atoms [i.e., κ = 1 (O) and κ = 2 (Be)]. Polarization in the zb crystal lattice is determined by point ion displacements from their equilibrium positions anticipating them to be rigid and non-polarizable. For zb BeO, $\omega_{j}\left(\vec{q}\right)$ are obtained in the harmonic approximation by solving the crystal Hamiltonian [67] using the following equation:

(1) $$H=\sum_{l\kappa \alpha }\frac{p_{\alpha }^{2}(l\kappa )}{2M_{\kappa }}+\Phi_{0}+\frac{1}{2}\sum_{l\kappa \alpha ,l^{\prime }\kappa^{\prime }\beta }\Phi_{\alpha \beta }(l\kappa ,l^{\prime }\kappa^{\prime })u_{\alpha }(l\kappa )u_{\beta }(l^{\prime }\kappa^{\prime })$$

where $u_{\alpha }(l\kappa )$ represents the displacement of the α-component for the $\kappa^{\mathrm{th}}$ (≡1, 2) type atom from equilibrium in l^th^ unit cell. The term $p_{\alpha }\left(l\kappa \right)$ signifies the corresponding components of its momentum. In zb crystals, the potential energy $\Phi_{\alpha \beta }\left(l\kappa ,l^{\prime }\kappa^{\prime }\right)$ can be divided into a short-range repulsive and long-range attractive Coulomb part as [67]

(2) $$\Phi_{\alpha \beta }\left(l\kappa ,l^{\prime }\kappa^{\prime }\right)=\Phi_{\alpha \beta }^{s}\left(l\kappa ,l^{\prime }\kappa^{\prime }\right)+{Z_{\kappa }Z_{\kappa^{\prime }}\Phi }_{\alpha \beta }^{C}(l\kappa ,l^{\prime }\kappa^{\prime })$$

with $Z_{\kappa }$e (≡$\;Z_{eff}$) being the charge on the $\kappa^{th}(\equiv 1,2)$ type ions.

The equations of motion in the harmonic approximation take the following form [67]:

(3) $$M_{\kappa }{\ddot{u}}_{\alpha }\left(l\kappa \right)=-\sum_{l^{\prime }\kappa^{\prime }\beta }\Phi_{\alpha \beta }(l\kappa ,l^{\prime }\kappa^{\prime })u_{\beta }(l^{\prime }\kappa^{\prime })$$

where the atomic displacement is expressed as a plane wave of the type:

(4) $$u_{\alpha }\left(l\kappa |\vec{q}j\right)=\frac{1}{\sqrt{M_{\kappa }}}e_{\alpha }(\kappa |\vec{q}j)e^{i[\vec{q}\vec{x}\left(l\kappa \right)-\omega_{j}\left(\vec{q}\right)t]};\;\mathrm{with}\;\alpha =x,y,z,$$

with t being the time; $\vec{x}\left(l\kappa \right)$ and $M_{\kappa }$ represent the position and mass of the (lκ) atom, respectively. Substituting Equation (4) into Equation (3), one can re-write the equations of motion as [67]

(5) $$\omega_{j}^{2}(\vec{q})e_{\alpha }(\kappa |\vec{q}j)=\sum_{\kappa^{\prime }\beta }D_{\alpha \beta }^{sC}(\kappa \kappa \prime |\vec{q})e_{\beta }(\kappa \prime |\vec{q}j);\kappa ,\kappa \prime =1,2$$

where $D_{\alpha \beta }^{sC}(\kappa \kappa \prime |\vec{q})\left[\equiv D_{\alpha \beta }^{s}(\kappa \kappa \prime |\vec{q})+D_{\alpha \beta }^{C}(\kappa \kappa \prime |\vec{q})\right]\;$represents the dynamical matrix comprising of the short-range $D_{\alpha \beta }^{s}(\kappa \kappa \prime |\vec{q}),$ and long-range Coulomb $D_{\alpha \beta }^{s}(\kappa \kappa \prime |\vec{q})$ interactions. For each mode $\omega_{j}(\vec{q})$, the components of its eigenvectors $e_{\alpha }(\kappa |\vec{q}j)$ in Equation (5) satisfy the familiar orthogonality (see Equation (6a)) [67]:

(6a) $$\sum_{\alpha \kappa }e_{\alpha }^{*}(\kappa |\vec{q}j)e_{\alpha }(\kappa |{\vec{q}j}^{\prime })=\delta_{jj^{\prime }},$$

and closure (see Equation (6b)) relations

(6b) $$\sum_{j}e_{\alpha }^{*}(\kappa \prime |\vec{q}j)e_{\beta }(\kappa |{\vec{q}j}^{\prime })=\delta_{\kappa \kappa^{\prime }}\delta_{\alpha \beta }.$$

#### 2.2.2. The Quasi Harmonic Approximation

In the harmonic approximation, the knowledge of IFCs at ambient P for zb BeO is required to obtain the phonon dispersions$\;\omega_{j}(\vec{q})$ (cf. Section 3). Inclusion of higher terms in Equation (1) makes the exact solutions of Equation (1) impossible. A QHA is adopted to calculate thermodynamical properties [84]. Simulations are made to study how the atomic vibrations (phonons) change with the temperature, T and volume V. In QHA, the interatomic potential is still terminated at the quadratic term, allowing for the definition of normal modes as in the harmonic approximation. The IFCs are now allowed to alter with a change of interatomic distance $a_{0}$. This means that the strength of force constants is modified by the variation of volume V or pressure P. Thus, one expects the phonon dispersions $\omega_{j}(\vec{q})$ and density of states DOS of the zb BeO material (cf. Section 3, Section 3.1 and Section 3.2) to shift with the change of V (or P), be it due to thermal expansion and/or by an externally applied stress (pressure, P).

#### 2.2.3. Interatomic Force Constants of zb BeO at P = 0 GPa

Systematic procedures [68] are followed to optimize IFCs at P = 0 GPa for zb BeO. Appropriate values of phonon frequencies at critical points (see Table 2) are used as the input while the elastic- $c_{ij}$ and lattice- $a_{0}$ constants are employed as constraints. To obtain IFCs (see Table 3) at P = 20 GPa, the values of ${\partial \omega }_{j}\left(\vec{q}\right)$/∂P from high-P RSS [96] or theoretical methods are used as the input with $\partial c_{ij}/\partial P$ elastic- and $\partial a_{0}/\partial P$ (cf. Section 3) as constraints. Once the set of force constants $A,B,C_{\kappa },D_{\kappa },E_{\kappa },F_{\kappa }$ and effective charge $Z_{eff}(\equiv Z_{\kappa }$e) are obtained for zb BeO at ambient pressure (P = 0) and high pressure (P = 20 GPa), it is straight forward to simulate $\omega_{j}\left(\vec{q}\right),$ one phonon DOS by using Equation (5).

### 2.3. Thermodynamical Properties

Application of isotropic P in solids causes the decrease (increase) of atomic distances (bonding interactions). These changes trigger variations in their phonon frequencies. The T- and/or P-dependent lattice parameter $a_{0}$ in a solid is commonly measured by HR-XRD [76,77]. In most semiconductors, the effect of P is known to produce stiffening of the longitudinal-, transverse-optical ($\omega_{LO},\;\omega_{TO}$) phonons and longitudinal acoustic ($\omega_{LA}$) modes while instigating softening in their transverse acoustic ($\omega_{TA}$) phonon branches. Pressure-induced stiffening/softening influences the phase space for phonon scattering and thus affects their thermal conductivity κ(T). Besides κ(T), the specific heat $C_{v}(T)$, thermal expansion coefficient $\alpha \left(T\right)\;$and Grüneisen parameters γ(T) are the other three most important characteristics of zb semiconductors [76,77]. These features have contributed to the fundamental understanding of lattice anharmonicity.

The impact of P on $C_{v}(T)$ is rather complex. Compression often leads to an increase in the vibrational frequencies. Therefore, one expects a shift of $C_{v}(T)$ peak to higher T. Debye temperature $\theta_{D}\left(T\right)\;$relates to the maximum vibrational frequency of a solid and can decrease at lower T due to softening of $\omega_{TA}$ modes [76,77]. The γ(T) describes anharmonicity of lattice vibrations and is related to the P dependence of phonon modes. Thermal expansion coefficient $\alpha \left(T\right)\;$describes how a material expands or contracts with the change of T and can be affected by P. To study $C_{v}\left(T\right)$, researchers have typically used a differential scanning calorimetry [76,77]. This study allows estimation of $C_{v}(T)$ based on the temperature change and heat absorbed by the crystal. The values of $\theta_{D}\left(T\right)$ are assessed from the experimental data of $C_{v}(T)$. Measurements of $\alpha \left(T\right)\;$are performed using a three-terminal capacitance dilatometer [76]. Except for the wz BeO, no $\alpha \left(T\right)$ results are known for the zb BeO. Thermodynamic traits of zb BeO help evaluate its use in thermal-management applications. In the absence of experimental data, one must study these traits theoretically (cf. Section 2.3) by using a realistic lattice dynamical model.

At a constant P, the α(T) of a material reflects the fractional change of its dimension (i.e., either length *l*, or volume V) per degree change in T. Thermal expansion coefficient can be calculated in QHA by minimizing Helmholtz free energy with respect to V, while considering T dependence of lattice constant $a_{0}$ and $\omega_{j}(\vec{q})$ [76,77]:

(7) $$\alpha =\frac{1}{3B_{0}}\sum_{\vec{q},j}C_{v}\left(\vec{q},j\right)\gamma_{j}\left(\vec{q}\right),$$

where $B_{0}$ is the bulk modulus. In Equation (7), the summation on the right-hand side is performed over the phonon modes$\;\left(\vec{q},j\right)$. At a constant V, the vibrational frequencies $\omega_{j}(\vec{q})$ of a material are assumed independent of T. The relative change of phonon frequencies $\frac{d\omega_{j}\left(\vec{q}\right)}{dV}$ with V (or P, $\frac{d\omega_{j}\left(\vec{q}\right)}{dP}$) is usually described by the mode Grüneisen dispersions (see Section 3.1 and Section 3.2) $\gamma_{j}\left(\vec{q}\right).$ This dimensionless quantity is defined as [76,77]:

(8) $$\gamma_{j}\left(\vec{q}\right)=-\frac{d\left(ln\omega_{j}\left(\vec{q}\right)\right)}{dlnV}=-\frac{V}{\omega_{j}\left(\vec{q}\right)}\frac{d\omega_{j}\left(\vec{q}\right)}{dV}=\frac{B_{0}}{\omega_{j}\left(\vec{q}\right)}\frac{d\omega_{j}\left(\vec{q}\right)}{dP}.$$

The above Equation (8) serves to quantify the vibrational anharmonicity. Positive sign of $\gamma_{j}\left(\vec{q}\right)\;$implies that the phonon frequencies are increasing with the decrease in V (or increase in P). This causes a positive coefficient of thermal expansion and allows dimensional stability of the material when exposed to the variations of T. This crucial property of the material is essential for applications requiring high thermal conductivity and electrical insulation. Low positive α(T) in a semiconductor helps minimize stress and potential damage caused by T fluctuation. The relevance of a positive $\alpha \left(T\right)$ for zb BeO will be discussed in Section 3.1 and Section 3.2.

Using $\gamma_{j}\left(\vec{q}\right),$ thermal Grüneisen parameter γ(T) can be obtained by using [76,77]

(9) $$\gamma \left(T\right)=\frac{\sum_{\vec{q},j}\gamma_{j}\left(\vec{q}\right)C_{v}\left(\vec{q},j\right)}{\sum_{\vec{q},j}C_{v}\left(\vec{q},j\right)},$$

where the contribution of each mode $\omega_{j}(\vec{q})$ to $\gamma \left(T\right)\;$is weighted in $C_{v}\left(\vec{q},j\right)$. The denominator in Equation (9) is $C_{v}\left(T\right),$ which takes the form [76,77].

(10) $$C_{v}(T)=k_{B}\sum_{\vec{q},j}{\left(\frac{\hbar \omega_{j}\left(\vec{q}\right)}{2k_{B}T}\right)}^{2}\frac{1}{{sinh}^{2}\left(\frac{\hbar \omega_{j}(\vec{q})}{2k_{B}T}\right)}.$$

Here, T is the temperature; $k_{B}$ and ħ are the Boltzmann and Planck constants, respectively. It is possible to express $C_{v}$ via the calculated $\omega_{j}(\vec{q})$ and DOS g(ω). Thus, an equivalent form of Equation (10) can be rewritten as [76,77]

(11) $$C_{v}(T)={Nrk}_{B}\int_{0}^{\infty }d\omega g(\omega )\left(\frac{\hbar }{k_{B}T}\right)\frac{exp\left(\frac{\hbar \omega_{j}(\vec{q})}{k_{B}T}\right)}{{\left[exp\left(\frac{\hbar \omega_{j}(\vec{q})}{k_{B}T}\right)-1\right]}^{2}}$$

By using Equations (9)–(11), the T-dependence on γ(T) are evaluated [76,77]:

(12) $$\gamma \left(T\right)=\frac{\sum_{\vec{q},j}\gamma_{j}\left(\vec{q}\right)C_{v}\left(\vec{q},j\right)}{C_{v}(T)}.$$

From the Debye’s equation, $C_{v}(T)$ can also be expressed as [76,77]

(13) $$C_{v}(T)={9rk}_{B}{\left(\frac{T}{\Theta_{D}(T)}\right)}^{3}\int_{0}^{\Theta_{D}(T)/T}\frac{x^{4}e^{x}}{{\left(e^{x}-1\right)}^{2}}dx,$$

In zb BeO, once the complete phonon dispersions $\omega_{j}\left(\vec{q}\right),DOS,$ Grüneisen dispersions $\gamma_{j}\left(\vec{q}\right)\;$(see Section 3.1, Section 3.1.1, Section 3.1.2, Section 3.2, Section 3.2.1 and Section 3.2.2) are systematically obtained for the wave vectors throughout the BZ. Using $\omega_{j}\left(\vec{q}\right),DOS,$ we have reported our calculated results of different thermodynamical characteristics including the Debye temperature $\Theta_{D}\left(T\right),Grüneisenparameter\gamma \left(T\right)$ and/or thermal expansion coefficient (see Section 3.1.1, Section 3.1.2, Section 3.2.1 and Section 3.2.2) $\alpha \left(T\right)$, etc.

## 3. Numerical Computations, Results and Discussions

In Section 2.2.3, we have obtained an optimized set of eleven IFCs for zb BeO material at ambient P = 0 GPa. Simulated P-dependent lattice and elastic constants (cf. Section 3.1) are necessary for assessing the force constants at higher P ≠ 0 GPa.

### 3.1. Pressure-Dependent Lattice and Elastic Constabts of zb BeO

By using Murnaghan equation of state (MEOS) with appropriate parameters (see Table 2) of zb BeO, we have calculated the P-dependent changes in $\frac{a}{a_{0}}\left[or\;\frac{V}{V_{0}}\right]$ [74]:

(14) $$\frac{a}{a_{0}}={\left[\frac{B_{0}^{\prime }}{B_{0}}P+1\right]}^{-\frac{1}{3B_{0}^{\prime }}}\;\mathrm{or}\;\frac{V}{V_{0}}={\left[\frac{B_{0}^{\prime }}{B_{0}}P+1\right]}^{-1/B_{0}^{\prime }},$$

where $B_{0}$ is the bulk modulus and $B_{0}^{\prime }$ is its pressure derivative.

**Figure 2:**
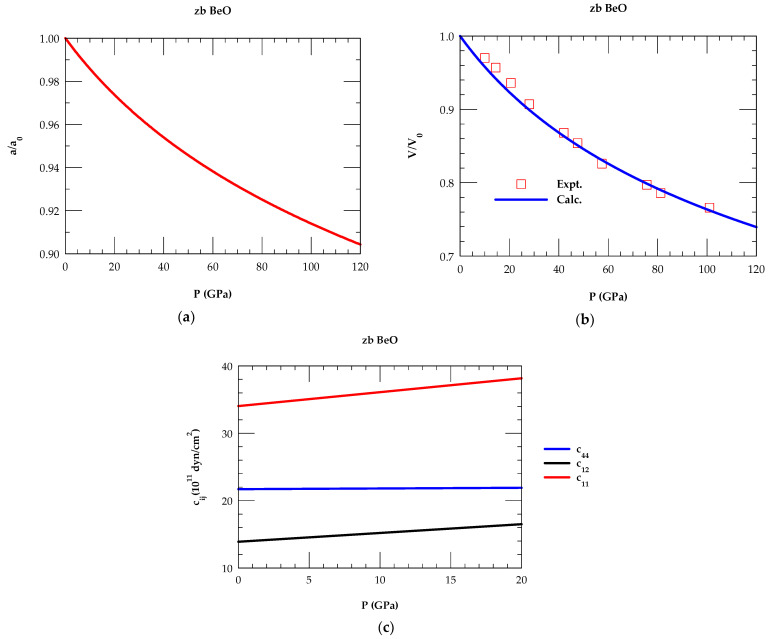
(**a**) Murnaghan’s equation of state is used to calculate lattice constant ratio $\frac{a}{a_{0}}$ as a function of P, (**b**) comparison of P-dependent volume ratio $\frac{V}{V_{0}}$ with the experimental data, (**c**) calculated variations of the elastic constants of $c_{11}$, $c_{12}$ and $c_{44}$ as a function of P, satisfying the mechanical stability conditions, viz., ($c_{11}$ − $c_{12}$) > 0, ($c_{11}$ + 2$c_{12}$) > 0, and $c_{44}$ > 0.

The results of P-dependent variations of $\frac{a}{a_{0}}$ and $\frac{V}{V_{0}}$, are displayed in Figure 2a,b respectively, for 0 < P < 120 GPa. Modifications in P-dependent elastic constants $c_{ij}$ displayed in Figure 2c revealed satisfaction of the mechanical stability conditions in zb BeO.

### 3.1.1. Interatomic Force Constants of zb BeO at P ≠ 0 GPa

To achieve IFCs for zb BeO at P ≠ 0, the P-dependent ∂ω/∂P phonons at a few critical points are used as the input while ∂a/∂P (see Figure 2a) and ∂c_ij_/∂P (see Figure 2c) are employed as constraints. Perusal of Table 3 has clearly indicated that the force constants of zb BeO changed significantly with P. These outcomes are consistent with similar results obtained earlier in compound semiconductors [69,70,71,72,73]. To appreciate the importance of two sets of IFCs, a linear interpolation scheme is considered [69,70]:

(15) $$a_{i}\left(P\neq 0\right)=a_{i}\left(P=0\right)+P\frac{da_{i}}{dP},$$

where $a_{i}$ (i = 1, 11) represents the values of force constants. By using Equation (15) and Table 3, one can calculate P-dependent IFCs at any desired P. This helped us in assessing P-dependent $\omega_{j}(\vec{q})$ (see Section 3.1.2) to comprehend thermodynamical (see Section 3.1.3) characteristics of zb BeO.

### 3.1.2. Phonon Dispersions and Density of States

**Figure 3:**
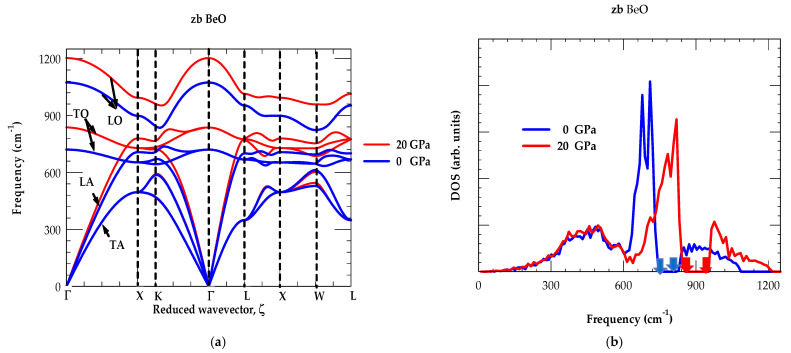
(**a**) Rigid-ion model calculations of phonon dispersions for zb BeO where blue and red color lines represent our results at ambient P = 0 GPa and P = 20 GPa, respectively. Black color arrows signify TA, LA, TO and LO phonon modes; (**b**) same as (**a**) but for the one phonon density of states, blue and red color arrows show a gap at high frequency at P = 0 GPa and P = 20 GPa.

For zb BeO, the phonon dispersions along high-symmetry directions (Γ → X → K → Γ → L → X → W → L) of the BZ are displayed in Figure 3a both at ambient P = 0 GPa (blue color lines) and P = 20 GPa (red color lines). Simulated results of one-phonon DOS $g\left(\omega \right),$ at P = 0 GPa and P = 20 GPa, are also reported in Figure 3b. In the absence of INS data at P = 0 GPa, the values of $\omega_{LO}$, $\omega_{TO}$, $\omega_{LA}$ and $\omega_{TA}$ are compared reasonably well with the ab initio calculation [66]. Like P = 0 GPa, the $\omega_{j}(\vec{q})$ and $g\left(\omega \right)\;$at P = 20 GPa (red color lines) exhibit identical features (Figure 3a,b). Unlike zb ZnO and other II-VI semiconductors, the RIM calculation in zb BeO has indicated atypical phonon features [94,95,96]. Our results at P = 20 GPa predict no appreciable changes in the $\omega_{TA}$ modes while other phonons $\omega_{LA}$, $\omega_{TO}$, $\omega_{LO}$ show shifts to higher frequencies. Similar outcomes are noticed in the one phonon DOS $g\left(\omega \right)$ (red color lines) with no visible change perceived in the $\omega_{TA}$ modes while high-frequency phonons shift upwards. The importance of this phonon mode behavior will be discussed next.

Both low $\omega_{TA}{,\omega }_{LA}$ and high frequency $\omega_{TO}$, $\omega_{LO}$ phonons influence the basic properties of zb BeO. Such traits impact on the material’s structural behavior, charge carrier dynamics and optical response. These features can also affect the performance of photonic devices. At low T, $\omega_{TA,LA}$ modes are the major carriers of heat and contribute to thermal conductivity κ. In BeO, the acoustic phonons interacting with electrons influence the scattering rates and change the overall electron transport operations. As the acoustic bands remain well-defined at high T, they can maintain the material’s structural integrity. High-frequency optical phonons in BeO play an important role in its electronic properties. They influence phenomena like phonon-assisted optical transitions as well as their response to electromagnetic radiation. The $\omega_{LO\left(\Gamma \right)}\;$ modes are crucial in the thermalization process of hot carriers for dissipation of excess heat which play vital roles in device performance. Splitting of $\omega_{LO\left(\Gamma \right)}-\omega_{TO\left(\Gamma \right)}\;$is a key feature of polar materials which arises from the high sensitivity of $\omega_{LO}$ phonons to acoustic warping of the intrinsic electric fields.

In high polar zb BeO, our RIM study has revealed large $\omega_{LO\left(\Gamma \right)}-\omega_{TO\left(\Gamma \right)}$ phonon splitting ~353 cm^−1^ at Γ point in the BZ. Moving away from Γ point, the $\omega_{LO}\left(\omega_{TO}\right)\;$modes exhibit high (low) dispersive behavior along the Γ →X, X → Γ and Γ → L directions (see Figure 3a). Most zb II–VI materials including zb ZnO show flatness of $\omega_{TA}$ branches. Unlike II–VI semiconductors, an unusual phonon behavior is noticed in zb BeO where the $\omega_{LA}\;$modes exhibit higher frequencies than $\omega_{TO}$ phonons (i.e., $\omega_{LA}>\omega_{TO})$. Accordingly, in DOS $g\left(\omega \right)$, the study has perceived no clear phonon gap between the acoustic and optical phonon branches (see Figure 3b). The results of one-phonon DOS $g\left(\omega \right)\;$disclose a broad band between 825 cm^−1^ and 1075 cm^−1^ with a minimum gap appearing in the frequency range of 744 cm^−1^ –825 cm^−1^ at P = 0 GPa (blue colored arrows), which shifts to higher frequency range of 850 cm^−1^–960 cm^−1^ at P = 20 GPa (red colored arrows).

Interestingly, identical trends are noted in zb BN and diamond C, for their $\omega_{j}\left(\vec{q}\right)$ [97,98,99,100,101]. This provides a strong support to our earlier assertion that the structural, elastic and vibrational characteristics of zb BeO exhibit similarities with super hard BN and C materials [70]. Moreover, in zb BeO the eigenvectors are linked to $\omega_{LO(X)}$ vibrations of lighter Be atom and heavier O oscillation to $\omega_{LA(X)}$ phonon [66]. In Table 4, the results of our RIM phonon frequencies at high critical points are compared reasonably well with the existing ab initio calculations [66]. At ambient and higher P, the $\omega_{j}(\vec{q})$ and $g\left(\omega \right)$ are used to simulate thermodynamical characteristics of zb BeO. The major impact of T is reported on different quantities such as $\Theta_{D}\left(T\right)$, $C_{v}\left(T\right),$(Section 3.1.3) $\gamma_{j}(\vec{q})$ and $\alpha \left(T\right)\;$(Section 3.2).

### 3.1.3. Debye Temperature and Specific Heat

**Figure 4:**
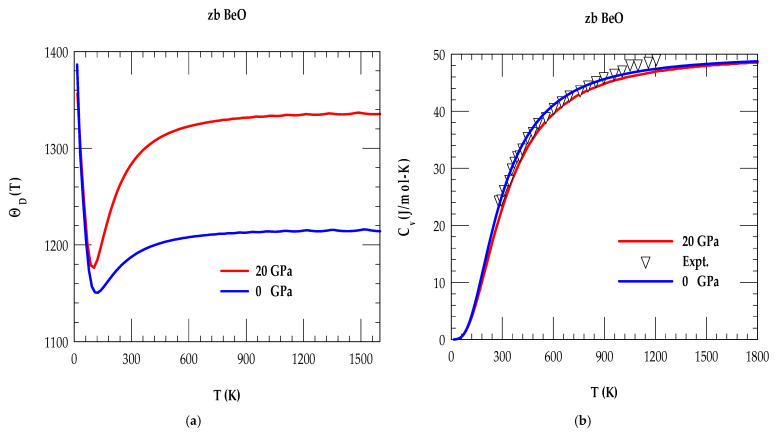
(**a**) Rigid-ion model calculations of the Debye temperature for the zinc blende BeO material as a function of T. The full blue- and red-color lines represent our calculated results at 1 atm or P = 0 GPa and P = 20 GPa, respectively. (**b**) Comparison of the rigid-ion model calculations of specific heat at constant volume for the zb BeO as a function of T with experimental data [80,81] (inverted black triangle). Solid blue- and red-color lines represent our calculated results at 1 atm or P = 0 GPa and P = 20 GPa, respectively.

In Figure 4a, we have displayed our RIM results of zb BeO for $\Theta_{D}\left(T\right)$ at P = 0 GPa (blue colored line) and P = 20 GPa (red colored lines) in the T range, 0 ≤ T ≤ 1600 K. Similar calculations are reported in Figure 4b for $C_{v}(T)$ for T between 0 ≤ T ≤ 1800 K at P = 0 GPa (blue colored line) and P = 20 GPa (red colored lines), respectively.

At P = 0 GPa, the RIM calculation of $\Theta_{D}\;$for the zb BeO material at nearly 0 K has provided a value of $\Theta_{D}\left(T\to 0\right)$ ~ 1390 K. It attained a minimum $\Theta_{D}^{min}\left(T\right)\;\sim \;1150\;K$ at ~ 124 K and reached a higher value at room temperature (RT) of $\Theta_{D}\left(297\right)$ ~ 1187 K, respectively. In the absence of P-dependent measurements of $\Theta_{D}$, the study for zb BeO at P = 20 GPa has predicted a slight decrease in $\Theta_{D}\left(T\to 0\right)$ ~1370 K, while attaining $\Theta_{D}^{min}\left(T\right)\;$∼1177 K at 93 K and achieving $\Theta_{D}\left(297\right)\;$~1291 K at RT, respectively. The simulated trends in the zb BeO material have agreed reasonably well with the behavior noticed in many compound semiconductors [69,70,71,72,73]. The rise of $\Theta_{D}\;\left(T\right)\;$at temperature (i.e., T > RT) can be attributed to the P-dependent stiffening of the high frequency $\omega_{LA}$, $\omega_{TO}$ and $\omega_{LO}$ phonon modes.

In Table 5, we have summarized our theoretical results by comparing them with the limited theoretical and/or experimental data for $\Theta_{D}\left(0\right)$, $\Theta_{D}^{min}\left(T\right)$, $\Theta_{D}\left(297\right),\;\Theta_{D}\left(HighT\right)\;$in (K) [6,80,81]. Similar comparison of systematic calculations for zb BeO is also made in Table 5 for the specific heat $C_{V}$(100), $C_{V}$(297), $C_{V}$(High T) in (J/mol-K) and linear thermal expansion coefficient α(T) in (10^−6^ K^−1^) [6,80,81] with the experimental data.

### 3.2. Pressure-Dependent Characteristics of zb BeO

By using DACs, many P-dependent Raman scattering studies are performed for comprehending the acoustical and optical phonons in several tetrahedrally coordinated elemental group IV and II–VI, III–V compound semiconductors [69,70,71,72,73,101,102]. Except for III–Ns and SiC, the existing data on most materials have confirmed [69,70,71,72,73,102] observing pressure-induced mode softening $\gamma_{TA(X,L)}\;$ of the zone edge transverse acoustical $\;\omega_{TA(X,L)}$ phonons. The P-induced stiffening is perceived, however, in every material for their zone edge longitudinal acoustical $\gamma_{LA(X,L)};$ $\omega_{LA(X,L)}$ and high frequency optical $\gamma_{LO,TO(X,L)};$ $\omega_{LO,TO(X,L)}$ phonons. Compression-induced softening of $\omega_{TA(X,L)}$ phonons in II–VI materials and zb ZnO have triggered a negative tension in their bonds due to increased repulsion of electron-charge overlaps. Such mode softening is responsible for attributing the negative thermal expansion coefficients $\alpha \left(T\right)\;$ in many II–VI semiconductors [100,101,102] including zb ZnO [94]. Absolutely no such experimental results of $\alpha \left(T\right)\;$ exist for zb BeO.

#### 3.2.1. Grüneisen Dispersions

In Table 4, we have reported our RIM results of zb BeO for the linear pressure coefficients $a_{j}^{P}$(≡ $\partial \omega_{j}(\vec{q})/\partial P$) and $\gamma_{j}\left(\vec{q}\right)\mathrm{at}\;a\;few\;critical\;points\;in\;the\;BZ$. The methodology outlined in Section 2.2 and Section 2.3 is adopted for simulating the $\vec{q}$-dependent $\gamma_{j}\left(\vec{q}\right)$. The Grüneisen constants $\gamma_{TA(X,L)}$ for zone edge $\omega_{TA(X,L)}\;modes$ revealed very small negative values (see Table 4) as compared to the large positive results noticed for high-frequency longitudinal acoustic $\omega_{LA}\;$and optical $\omega_{LO},\omega_{TO}\;$phonons. For zb BeO, the T-dependent simulations of $\alpha \left(T\right)$ reported in Section 3.2.2 will be compared/contrasted and discussed with the existing experimental [80,81] and theoretical [82,83] data.

**Figure 5:**
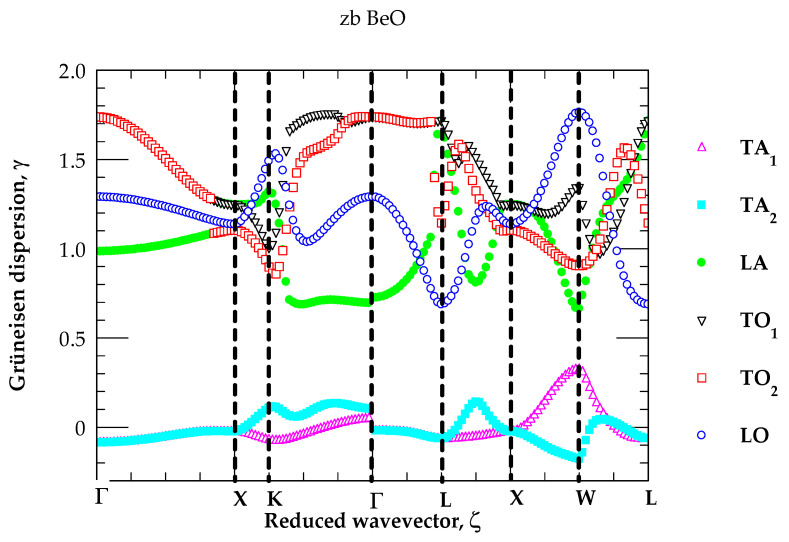
Rigid-ion model calculations of mode Grüneisen dispersions $\gamma_{j}\left(\vec{q}\right)\;$ of the zinc blende BeO along high symmetry direction (Γ → X → K → Γ → L → X → W → L) of the BZ. Different colored symbols on the right-hand side of the graph represent the $\gamma_{j}\;$ for optical and acoustical modes.

In Figure 5, we have displayed our simulated results of mode Grüneisen dispersions $\gamma_{j}\left(\vec{q}\right)$ for zb BeO along high symmetry directions. The results are consistent with the selected values listed in Table 4 at a few high critical points. These features have attested some important facts that in zb BeO, the $\gamma_{TA(X,L)}$ of $\omega_{TA\left(X,L\right)\;}$phonons exhibit much smaller negative values as compared to the large positive values of $\gamma_{j}\left(\vec{q}\right)$ for the high-frequency $\omega_{LA(X,L)},\omega_{LO,TO(X,L)}$ modes. The impact of these$\;\vec{q}$-dependent $\gamma_{j}\left(\vec{q}\right)\;$ on linear thermal expansion coefficient $\alpha \left(T\right)\;$ of zb BeO is reported in Section 3.2.2.

#### 3.2.2. Thermal Expansion Coefficient

**Figure 6:**
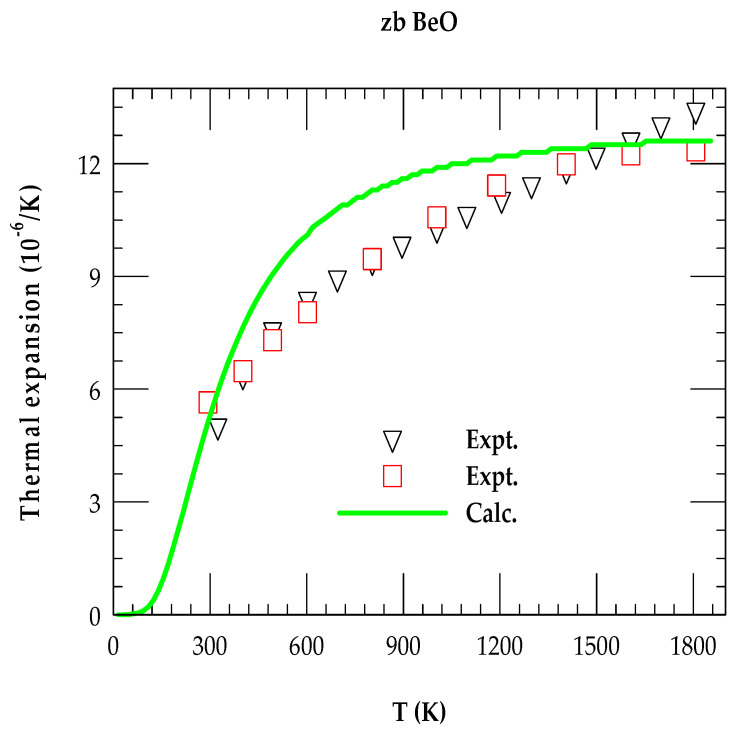
For zinc blende BeO, the comparison of rigid-ion model calculations (green color line) of linear thermal expansion coefficient α(T) as a function of T with available experimental data. The experimental results shown by the red color open squires are taken from Slack and Bartram [80] while black-colored inverted triangles are from Kozlovskii et al. [81].

The T-dependent α(T) is simulated for zb BeO by carefully incorporating $\gamma_{j}\left(\vec{q}\right)$ and $C_{v}(T)$ in Equations (7)–(9). The RIM results displayed in Figure 6 for 0 ≤ T ≤ 1900 K are compared with the limited experimental data [80,81].

Unlike zb ZnO which showed negative thermal expansion [73] at low temperature, our RIM study of zb BeO has exhibited positive α(T) in the entire T range. Theoretical study has provided (green color line) a reasonably good comparison with the existing experimental data of Slack and Bartram [80] (open red square) and Kozlovskii et al. [81] (open inverted black triangle). Obviously, the RIM simulations have established an important fact that the sum of positive high-frequency Grüneisen $modes\;(\gamma_{LO,TO(X,L)};$ $\omega_{LO,TO(X,L)})$ in Equation (7) are dominated over the very small negative values of $\gamma_{TA(X,L)}$. Thus, at low T, the study has offered no negative (see Figure 6) values of α(T).

## 4. Concluding Remarks

Understanding the electronic, structural and thermal properties of a solid requires precise knowledge of its vibrational characteristics [77]. In the absence of INS data for zb BeO, MgO, and CdO, theoretical simulations of lattice dynamics and thermodynamical properties have played crucial roles in solid-state physics, materials science and electronics. Among the II–Os, the wide bandgap BeO has offered exceptional thermal conductivity, chemical stability and high melting temperature. The material is considered valuable for designing optoelectronics, power electronics and thermal management systems. To develop LDH-based advanced flexible micro- and nano-electronics, the knowledge of phonon dispersion $\omega_{j}\left(\vec{q}\right)$ and thermal properties [e.g., $\Theta_{D}\left(T\right),\;C_{V}\left(T\right),\;\kappa \left(T\right),$ γ(T), α(T)] of zb BeO is crucial. By adopting a realistic RIM, we have reported results of our methodical studies at ambient and high-pressure P to comprehend its structural, phonon and thermodynamical properties. At ambient P = 0 GPa, the study has provided accurate $\omega_{j}\left(\vec{q}\right)\;$in good agreement with the first-principle calculations [66]. At higher P = 20 GPa, the predictions are made for the $\omega_{j}\left(\vec{q}\right)$, as well as T-dependent $\Theta_{D}\left(T\right)\;$and $C_{V}\left(T\right)$.

Unlike zb ZnO, our RIM study has exhibited atypical phonon and thermodynamical properties for the zb BeO material. In zb BeO, the $\omega_{LA}\;$mode frequencies along Γ → X and Γ → L directions are higher than the $\omega_{TO}$ phonons. Vibrational characteristics in zb ZnO are analogous to the conventional II–VI compound semiconductors. Like zb BeO, similar unusual trends in the phonon characteristics are also noticed in zb BN and diamond C materials [96,97,98,99,100]. These features have provided strong corroboration to our earlier assertion that the structural, elastic and vibrational traits of zb BeO exhibit similarities to the super hard BN and C materials [70]. In zb BeO, the acoustic phonons are the major heat carriers. In $\omega_{LA}$ modes, the Be and O atoms vibrate in the same direction as the wave propagation. However, in the $\omega_{TO}$ phonons, the atoms move perpendicular to the wave propagation. As $\omega_{LA}>\omega_{TO}$, one expects $\omega_{LA}$ modes to carry more energy than the $\omega_{TO}$ phonons. This could lead to increased scattering rates and possibly lower the thermal conductivity of zb BeO. The interplay between $\omega_{LA}$ and $\omega_{TO}$ modes and their respective scattering mechanisms is a challenging issue and requires further studies for accurately predicting and understanding thermal transport properties in zb BeO.

As compared to zb ZnO, the zone edge Grüneisen constants $\gamma_{TA(X,L)}$ for $\omega_{TA(X,L)}$ modes in zb BeO material have revealed very small negative values. Thus, our systematic calculations in the zb ZnO have exhibited negative α(T) at low T while positive results are predicted for zb BeO material in the entire 0 ≤ T ≤ 1900 K range. In semiconductors, the mode-softening of $\gamma_{TA(X,L)}$ triggers negative tension in their atomic bonding due to increased repulsion of electron-charge overlaps to cause phase transitions. Both the zb BeO, ZnO and their Be_x_Zn_1−x_O alloys [94,95] are known to play important roles for managing heat dissipation in high-power electronics, optoelectronic devices, and flexible nano modules operating at varying temperatures which specifically require precise dimensional stability. Controlling and designing novel materials with a specific value of α(T) is essential for managing heat, especially in miniaturized high-density devices for high-speed integrated electronics. By combining zb BeO and ZnO materials with positive and negative thermal expansion, it is quite likely to create LDH-based structures with tailored α(T) even for achieving near-zero value.

## Figures and Tables

**Table 1:** The transition pressures and relative volume changes for wurtzite BeO to rock-salt (B4 → B1), zinc blende to rock salt (B3 → B1), and wurtzite to zinc blende (B4 → B3) structures.

Material	$P_{t}$ ^(a)^$\;\mathrm{and}∆V_{t}$$/V_{0}$	B_4_ → B_1_^(a)^	Others ^(a)^	B_3_ → B_1_^(a)^	Others ^(a)^	B_4_ → B_3_^(a)^	Others ^(a)^
BeO	$P_{t}$ (GPa)	137.3 147.0	21.7; 40.0; 95.0	139.0 95.0	94.0–147.0 94.0–96.0	74.0 91.0	62–91
	$∆V_{t}$ (%)	11.20	11.0				

^(a)^ References [84,85,86,87,88,89,90,91,92,93] (and references cited therein).

**Table 2:** Critical-point phonon frequencies (cm^−1^) of zb ZnO and BeO, lattice constants$\;a_{o}$ in (Å) elastic constants $c_{ij}\;$10^11^ dyn/cm^2^, transition pressure P_t_ (GPa). These parameters are used for evaluating the rigid-ion model (RIM) parameters at ambient pressure (cf. Section 3).

	zb ZnO (our) ^(a)^	Others ^(b)^	zb BeO (our) ^(a)^	Others ^(d)^	Raman ^(c)^
$\omega_{LO\left(\Gamma \right)}$	558	525 ^(c)^	1074	1060	1093.7
$\omega_{TO\left(\Gamma \right)}$	403	403 ^(c)^	721	683	682–704
$\omega_{LO(X)}$	551	555 ^(c)^	899	900	
$\omega_{TO(X)}$	487	444 ^(c)^	653	655	
$\omega_{LA(X)}$	269	268 ^(c)^	707	708	
$\omega_{TA(X)}$	128	80 ^(c)^	496	493	
$\omega_{LO(L)}$	561		953	902	
$\omega_{TO(L)}$	443		669	663	
$\omega_{LA(L)}$	264		701	702	
$\omega_{TA(L)}$	93		349	310	
$a_{o}$	4.504	4.520–4.666	3.81	3.72–3.83	
$c_{11}$	19.19	15.1–19.3	34.2	34.2	
$c_{12}$	14.79	11.06–15.8	13.9	13.9–14.8	
$c_{44}$	7.34	7.4–12.8	21.7	20.8–21.7	
B_0_	162.6	128.8–177.3	207	201–229	
$B_{0}^{\prime }$	4.0	3.3–4.3	3.7	3.65–3.96	

^(a)^ Our ^(b)^ References. [67] ^(c)^ Raman [48,96] ^(d)^ Others [47,49,66].

**Table 3:** Optimized set of rigid-ion model parameters (10^5^ dyn/cm) for zb BeO at P = 0 and P = 20 GPa in the notation of Reference. [67]. The term Z_eff_ is the effective charge.

RIM ^(a)^	zb BeO	
Parameters	P = 0 GPa	P = 20 GPa
A	−0.62022	−0.806
B	−0.55000	−0.74
C_1_	−0.06650	−0.0715
C_2_	−0.09300	−0.102
D_1_	−0.04144	−0.02497
D_2_	−0.14900	−0.1634
E_1_	−0.10000	−0.18
E_2_	0.04000	0.04
F_1_	0.15500	0.218
F_2_	−0.12500	−0.106
Z_eff_	1.0133	1.056

^(a)^ Reference. [67].

**Table 4:** Comparison of critical point phonon frequencies (cm^−1^) at P = 0, for zb BeO using a rigid-ion model (RIM) with estimated ab initio calculation values. The RIM phonons at P = 20 GPa and linear pressure coefficients $a_{j}^{P}$ (cm^−1^/GPa) and Grüneisen parameters $\gamma_{j}\left(\vec{q}\right)\;$are also reported.

Modes zb BeO	Our RIM ^(a)^ P = 0 GPa	*Ab* initio Calc.^(b)^ P = 0 GPa	Our RIM ^(a)^ P = 20 GPa	$a_{j}^{P}=\frac{\partial \omega_{j}}{\partial P}$ RIM ^(a)^	$\gamma_{j}$ RIM ^(a)^
$\omega_{LO\left(\Gamma \right)}$	1074	1060	1201	6.35	1.29
$\omega_{TO\left(\Gamma \right)}$	721	683	836	5.75	1.74
$\omega_{LO(X)}$	899	900	993	4.7	1.14
$\omega_{TO(X)}$	653	655	730	3.85	1.25
$\omega_{LA(X)}$	707	708	779	3.6	1.1
$\omega_{TA(X)}$	496	493	494	−0.10	−0.06
$\omega_{LO(L)}$	953	902	1013	3	1.14
$\omega_{TO(L)}$	669	663	775	5.3	1.72
$\omega_{LA(L)}$	701	702	777	3.8	0.7
$\omega_{TA(L)}$	349	310	347	−0.10	−0.06

^(a)^ Our ^(b)^ Reference. [66] estimated values.

**Table 5:** For zb BeO, the RIM calculations of Debye temperature $\Theta_{D}\left(0\right)$, $\Theta_{D}^{min}\left(T\right)$, $\Theta_{D}\left(297\right)\;$in (K), $C_{V}$(297) in (J/mol-K) and α(T) in (10^−6^ K^−1^) are compared with the existing experimental and/or theoretical data from the literature.

zb BeO Quantity	RIM, P = 0 GPa ^(a)^	Others ^(b)^	$\alpha \left(T\right)$ Others ^(c)^	$\alpha \left(T\right)$ Others ^(d)^	RIM, P = 20 GPa ^(a)^
$\Theta_{D}\left(0\right)$	1390	1270; 1280			1370
$\Theta_{D}^{\min}\left(T\right)$	1150 @ 124 K				1177 @ 93 K
$\Theta_{D}\left(297\right)$	1187				1291
$\Theta_{D}\left(\mathrm{High}\;T\right)$	1214 @ 1000 K				1335 @ 1850 K
$C_{V}$(100)	3.17				2.67
$C_{V}$(297)	24.78	25.51–26.11			22.5
$C_{V}$(High T)	48.83 @ 1850 K	48.72 @ 1150 K			48.7 @ 1850 K
$\alpha \left(293\right)$	5.12		5.65 @ 300	4.99 @ 293	
$\alpha \left(401\right)$	7.64		6.48 @ 400	6.33 @ 373	
$\alpha \left(509\right)$	9.24		7.30 @ 500	7.55 @ 473	
$\alpha \left(1606\right)$	12.5		12.25 @ 1600	12.60 @ 1573	

^(a)^ Our, ^(b)^ Reference. [6], ^(c)^ Reference. [80], ^(d)^ Reference. [81].

## Data Availability

The original contributions presented in this study are included in the article. Further inquiries can be directed to the corresponding author.

## References

[1] Sharma D.K., Shukla S., Sharma K.K., Kumar V. (2022). A review on ZnO: Fundamental properties and applications. Mater. Today Proc..

[2] Pushpalatha C., Suresh J., Gayathri V.S., Sowmya S.V., Augustine D., Alamoudi A., Zidane B., Albar N.H.M., Patil S. (2022). Zinc Oxide Nanoparticles: A Review on Its Applications in Dentistry, Nanoparticles: A Review on Its Applications in Dentistry. Front. Bioeng. Biotechnol..

[3] Borysiewicz M.A. (2019). ZnO as a Functional Material, a Review. Crystals.

[4] Pearton S., Norton D., Ip K., Heo Y., Steiner T. (2003). Recent progress in processing and properties of ZnO. Superlattices Microstruct..

[5] Schmidt-Mende L., MacManus-Driscoll J.L. (2007). ZnO nanostructures, defects, and devices. Mater. Today.

[6] Özgür Ü., Alivov Y.I., Liu C., Teke A., Reshchikov M.A., Doğan S., Avrutin V., Cho S.-J., Morkoç H. (2005). A comprehensive review of ZnO materials and devices. J. Appl. Phys..

[7] Sashin V.A., Bolorizadeh M.A., Kheifets A.S., Ford M.J. (2003). Electronic Band Structure of Beryllium Oxide. J. Phys. Condens. Matter.

[8] Subramanian M.A., Shannon R.D., Chai B.H.T., Abraham M.M., Wintersgill M.C. (1989). Dielectric Constants of BeO, MgO, and CaOUsing the Two-Terminal Method. Phys. Chem. Miner..

[9] Yim K., Yong Y., Lee J., Lee K., Nahm H.-H., Yoo J., Lee C., Hwang C.S., Han S. (2015). Novel High-κ Dielectrics for Next-Generation Electronic Devices Screened by Automated Ab Initio Calculations. NPG Asia Mater..

[10] Yum J.H., Akyol T., Lei M., Ferrer D.A., Hudnall T.W., Downer M., Bielawski C.W., Bersuker G., Lee J.C., Banerjee S.K. (2012). Electrical and Physical Characteristics for Crystalline Atomic layer Deposited Beryllium Oxide Thin Film on Si and GaAs Substrates. Thin Solid Film..

[11] Yum J.H., Akyol T., Ferrer D.A., Lee J.C., Banerjee S.K., Lei M., Downer M., Hudnall T.W., Bielawski C.W., Bersuker G. (2011). Comparison of the Self-Cleaning Effects and Electrical Characteristics of BeO and Al_2_O_3_ Deposited as an Interface Passivation Layer on GaAs MOS Devices. J. Vac. Sci. Technol. A.

[12] Camarano D.M., Mansur F.A., Santos A.M.M., Ribeiro L.S., Santos A. (2019). Thermal Conductivity of UO2–BeO–Gd2O3 Nuclear Fuel Pellets. Int. J. Thermophys..

[13] Chandramouli D., Revankar S.T. (2014). Development of Thermal Models and Analysis of UO2-BeO Fuel during a Loss of Coolant Accident. Int. J. Nucl. Energy.

[14] Chen S., Yuan C. (2020). Neutronic Study of UO2-BeO Fuel with Various Claddings. Nucl. Mater. Energy.

[15] Garcia C.B., Brito R.A., Ortega L.H., Malone J.P., McDeavitt S.M. (2017). Manufacture of a UO2-Based Nuclear Fuel with Improved Thermal Conductivity with the Addition of BeO. Metall. Mater. Trans. E.

[16] Nicolay S., Fay S., Ballif C. (2009). Growth Model of MOCVD Polycrystalline ZnO. Cryst. Growth Des..

[17] Zhang J., Cui X., Shi Z., Wu B., Zhang Y., Zhang B. (2014). Nucleation and growth of ZnO films on Si substrates by LP-MOCVD. Superlattices Microstruct..

[18] Youdou Z., Shulin G., Jiandong Y., Wei L., Shunmin Z., Feng Q., Liqun H., Rang Z., Yi S. MOCVD Growth and Properties of ZnO and Zn_l-x_,Mg_x_O Films. Proceedings of the Sixth Chinese Optoelectronics Symposium.

[19] Kadhim G.A. (2024). Study of the Structural and Optical Traits of In:ZnO Thin Films Via Spray Pyrolysis Strategy: Influence of laser Radiation Change in Different Periods. AIP Conf. Proc..

[20] Wei X.H., Li Y.R., Zhu J., Huang W., Zhang Y., Luo W.B., Ji H. (2007). Epitaxial properties of ZnO thin films on SrTiO_3_ substrates grown by laser molecular beam epitaxy. Appl. Phys. Lett..

[21] Opel M., Geprags S., Althammer M., Brenninger T., Gross R. (2014). Laser molecular beam epitaxy of ZnO thin films and heterostructures. J. Phys. D Appl. Phys..

[22] Chauveau J.-M., Morhain C., Teisseire M., Laugt M., Deparis C., Zuniga-Perez J., Vinter B. (2009). (Zn, Mg)O/ZnO-based heterostructures grown by molecular beam epitaxy on sapphire: Polar vs. non-polar. Microelectron. J..

[23] Peltier T., Takahashi R., Lippmaa M. (2014). Pulsed laser deposition of epitaxial BeO thin films on sapphire and SrTiO3. Appl. Phys. Lett..

[24] Triboulet R., Perrière J. (2003). Epitaxial growth of ZnO films. Prog. Cryst. Growth Charact. Mater..

[25] Yıldırım Ö.A., Durucan C. (2010). Synthesis of zinc oxide nanoparticles elaborated by microemulsion method. J. Alloys Compd..

[26] Mao Y., Li Y., Zou Y., Shen X., Zhu L., Liao G. (2019). Solvothermal synthesis and photocatalytic properties of ZnO micro/ nanostructures. Ceram. Int..

[27] Araujo E.A., Nobre F.X., Sousa G.d.S., Cavalcante L.S., Santos M.R.M.C., Souza F.L., de Matos J.M.E. (2017). Synthesis, growth mechanism, optical properties and catalytic activity of ZnO microcrystals obtained via hydrothermal processing. RSC Adv..

[28] Brown R.A., Evans J.E., Smith N.A., Tarat A., Jones D.R., Barnett C.J., Maffeis T.G.G. (2013). The effect of metal layers on the morphology and optical properties of hydrothermally grown zinc oxide nanowires. J. Mater. Sci..

[29] Horio Y., Yuhara J., Takakuwa Y., Ogawa S., Abe K. (2018). Polarity identification of ZnO (0001) surface by reflection high-energy electron diffraction. Jpn. J. Appl. Phys..

[30] Chen Y., Bagnall D., Yao T. (2000). ZnO as a novel photonic material for the UV region. Mater. Sci. Eng. B.

[31] Huang M.R.S., Erni R., Lin H.-Y., Wang R.-C., Liu C.-P. (2011). Characterization of wurtzite ZnO using valence electron energy loss spectroscopy. Phys. Rev. B.

[32] Kaida T., Kamioka K., Ida T., Kuriyama K., Kushida K., Kinomura A. (2014). Rutherford backscattering and nuclear reaction analyses of hydrogen ion-implanted ZnO bulk single crystals. Nucl. Instrum. Methods Phys. Res. B.

[33] Ismail M.A., Taha K.K., Modwi A., Khezami L. (2018). ZnO Nanoparticles: Surface and X-ray profile analysis. J. Ovonic Res..

[34] Mohan A.C., Renjanadevi B. (2016). Preparation of Zinc Oxide Nanoparticles and its Characterization Using Scanning Electron Microscopy (SEM) and X-Ray Diffraction (XRD). Procedia Technol..

[35] Martínez-Tomás M.C., Hortelano V., Jiménez J., Wang B., Muñoz-Sanjosé V. (2013). High resolution X-ray diffraction, X-ray multiple diffraction and cathodoluminescence as combined tools for the characterization of substrates for epitaxy: The ZnO case. CrystEngComm.

[36] Chao L.-C., Yang S.-H. (2007). Growth and Auger electron spectroscopy characterization of donut-shaped ZnO nanostructures. Appl. Surf. Sci..

[37] Ni H., Li X. (2006). Young’s modulus of ZnO nanobelts measured using atomic force microscopy and nanoindentation techniques. Nanotechnology.

[38] Kirmse H., Sparenberg M., Zykov A., Sadofev S., Kowarik S., Blumstengel S. (2016). Structure of p-Sexiphenyl Nanocrystallites in ZnO Revealed by High Resolution Transmission Electron Microscopy. Cryst. Growth Des..

[39] Li X., Cheng S., Deng S., We X., Zhu J., Che Q. (2017). Direct Observation of the Layer-by Layer Growth of ZnO Nanopillar by In situ High Resolution Transmission Electron Microscopy. Sci. Rep..

[40] Raouf D. (2013). Synthesis and photoluminescence characterization of ZnO nanoparticles. J. Lumin..

[41] Saadatkia P., Ariyawansa G., Leedy K.D., Look D.C., Boatner L.A., Selim F.A. (2016). Fourier Transform Infrared Spectroscopy Measurements of Multi-phonon and Free-Carrier Absorption in ZnO. J. Electron. Mater..

[42] Keyes B.M., Gedvilas L.M., Li X., Coutts T.J. (2005). Infrared spectroscopy of polycrystalline ZnO and ZnO:N thin films. J. Cryst. Growth.

[43] Damen T.C., Porto S.P.S., Tell B. (1966). Raman Effect in Zinc Oxide. Phys. Rev..

[44] Calleja J.M., Cardona M. (1977). Resonant raman scattering in ZnO. Phys. Rev. B.

[45] Manjon F.J., Syassen K., Lauck R. (2002). Effect of pressure on phonon modes in wurtzite zinc oxide. High Press. Res..

[46] Kokila A., Jagannatha Reddy M.K., Nagabhushana H., Rao J.L., Shivakumara C., Nagabhushana B.M., Chakradhar R.P.S. (2011). Combustion synthesis, characterization and Raman studies of ZnO nano powders. Spectrochim. Acta Part A.

[47] Serrano J., Manjón F.J., Romero A.H., Ivanov A., Cardona M., Lauck R., Bosak A., Krisch M. (2010). Phonon dispersion relations of zinc oxide: Inelastic neutron scattering and ab initio calculations. Phys. Rev. B.

[48] Serrano J., Romero A.H., Manjo’n F.J., Lauck R., Cardona M., Rubio A. (2004). Pressure dependence of the lattice dynamics of ZnO: An ab initio approach. Phys. Rev. B.

[49] Serrano J., Manjón F.J., Romero A.H., Ivanov A., Lauck R., Cardona M., Krisch M. (2007). The phonon dispersion of wurtzite-ZnO revisited. Phys. Status Solidi (B).

[50] Bohórquez C., Bakkali H., Delgado J.J., Blanco E., Herrera M., Domínguez M. (2022). Spectroscopic Ellipsometry Study on Tuning the Electrical and Optical Properties of Zr-Doped ZnO Thin Films Grown by Atomic Layer Deposition. ACS Appl. Electron. Mater..

[51] Bhandari K.P., Sapkota D.R., Ramanujam B. (2024). Spectroscopic-ellipsometry study of the optical properties of ZnO nanoparticle thin films. MRS Commun..

[52] Chibueze T.C. (2021). Ab initio study of mechanical, phonon and electronic Properties of cubic zinc-blende structure of ZnO. Niger. Ann. Pure Appl. Sci..

[53] Zafar M., Ahmed S., Shakil M., Choudhary M.A. (2014). First-principles calculations of structural, electronic, and thermodynamic properties of ZnxO1–xSx alloys. Chin. Phys. B.

[54] Yu Y., Zhou J., Han H., Zhang C., Cai T., Song C., Gao T. (2009). Ab initio study of structural, dielectric, and dynamical properties of zinc-blende ZnX (X = O, S, Se, Te). J. Alloys Compd..

[55] Singh J., Jain V.K. (2023). Structural, Electronic and Optical Properties of ZnO Material Using First Principle Calculation. J. Polym. Compos..

[56] Mohammadi A.S., Baizaee S.M., Salehi H. (2011). Density Functional Approach to Study Electronic Structure of ZnO Single Crystal. World Appl. Sci. J..

[57] Charifi Z., Baaziz H., Reshak A.H. (2007). Ab-initio investigation of structural, electronic and optical properties for three phases of ZnO compound. Phys. Status Solidi (B).

[58] Qing X., Zhang C., Gong J., Chen S. (2021). Ab initio study of photoelectric properties in ZnO transparent conductive oxide. Vacuum.

[59] Song H.F., Liu H.F., Tian E. (2007). Structural and thermodynamic properties of hexagonal BeO at high pressures and temperatures. J. Phys. Condens. Matter.

[60] Bocharov D., Pudza I., Klementiev K., Krack M., Kuzmin A. (2021). Study of High-Temperature Behaviour of ZnO by Ab Initio Molecular Dynamics Simulations and X-ray Absorption Spectroscopy. Materials.

[61] Bachmann M., Czerner M., Edalati-Boostan S., Heiliger C. (2012). Ab initio calculations of phonon transport in ZnO and ZnS. Eur. Phys. J. B.

[62] Wang Z., Wang F., Wang L., Jia Y., Sun Q. (2013). First-principles study of negative thermal expansion in zinc oxide. J. Appl. Phys..

[63] Ren D., Xiang B., Gao Y., Hu C., Zhang H. (2017). Ab initio study of lattice instabilities of zinc chalcogenides ZnX (X=O, S, Se, Te) induced by ultrafast intense laser irradiation. AIP Adv..

[64] Calzolari A., Nardelli M.B. (2013). Dielectric properties and Raman spectra of ZnO from a first principles finite-differences/finite-fields approach. Sci. Rep..

[65] Liu J., Allen P.B. (2018). Internal and external thermal expansions of wurtzite ZnO from first principles. Comput. Mater. Sci..

[66] Duman S., Sütlü A., Bağcı S., Tütüncü H.M., Srivastava G.P. (2009). Structural, elastic, electronic, and phonon properties of zincblende and wurtzite BeO. J. Appl. Phys..

[67] Kunc K. (1973–1974). Dynamique de réseau de composés ANB8-N présentant la structure de la blende. Ann. Phys..

[68] Talwar D.N. (2024). Computational phonon dispersions structural and thermodynamical characteristics of novel C-based XC (X = Si, Ge and Sn) materials. Next Mater..

[69] Talwar D.N., Vandevyver M. (1990). Pressure-dependent phonon properties of III-V compound semiconductors. Phys. Rev. B.

[70] Talwar D.N., Becla P. (2025). Microhardness, Young’s and Shear Modulus in Tetrahedrally Bonded Novel II-Oxides and III-Nitrides. Materials.

[71] Talwar D.N., Sherbondy J.C. (1995). Thermal expansion coefficient of 3C–SiC. Appl. Phys. Lett..

[72] Talwar D.N. (2002). Phonon excitations and thermodynamic properties of cubic III nitrides. Appl. Phys. Lett..

[73] Talwar D.N. (2023). Pressure-dependent mode Grüneisen parameters and their impact on thermal expansion coefficient of zinc-blende InN. J. Mater. Sci..

[74] Murnaghan F.D. (1944). The Compressibility of Media under Extreme Pressures. Proc. Natl. Acad. Sci. USA.

[75] Mori Y., Niiya N., Ukegawa K., Mizuno T., Takarabe K., Ruoff A.L. (2004). High-pressure X-ray structural study of BeO and ZnO to 200 GPa. Phys. Status Solidi (B).

[76] Boer K.W., Pohl U.W. (2014). Phonon-Induced Thermal Properties, Semiconductor Physics.

[77] Morkoç H., Özgür Ü. (2009). Zinc Oxide: Fundamentals, Materials and Device Technology.

[78] Yates B., Coopers R.F., Kreitman M.M. (1971). Low-Temperature Thermal Expansion of Zinc Oxide. Vibrations in Zinc Oxide and Sphalerite Zinc Sulfide. Phys. Rev. B.

[79] Ibach H. (1969). Thermal Expansion of Silicon and Zinc Oxide (II). Phys. Status Solidii.

[80] Slack G.A., Bartram S.F. (1975). Thermal expansion of some diamondlike crystals. J. Appl. Phys..

[81] Kozlovskii Y.M., Stankus S.V. (2014). Thermal expansion of beryllium oxide in the temperature interval 20–1550 °C. High Temp..

[82] Luo F., Cheng Y., Cai L.C., Chen X.R. (2013). Structure and thermodynamic properties of BeO: Empirical corrections in the quasi-harmonic approximation. J. Appl. Phys..

[83] Wdowik U.D. (2010). Structural stability and thermal properties of BeO from the quasi-harmonic approximation. J. Phys. Condens. Matter.

[84] Sahariah M.B., Ghosh S. (2010). Dynamical stability and phase transition of BeO under pressure. J. Appl. Phys..

[85] Yu B.R., Yang J.W., Guo H.Z., Ji G.F., Chen X.R. (2009). Phase transition and elastic properties of BeO under pressure from first-principles calculations. Phys. B.

[86] Zhang Q.L., Zhang P., Song H.F., Liu H.F. (2008). Mean-field potential calculations of high-pressure equation of state for BeO. Chin. Phys. B.

[87] Bosak A., Schmalzl K., Krisch M., van Beek W., Kolobanov V. (2008). Lattice dynamics of beryllium oxide: Inelastic X-ray scattering and ab initio calculations. Phys. Rev. B.

[88] Sahariah M.B., Ghosh S. (2008). Ab initio calculation of lattice dynamics in BeO. J. Phys. Condens. Matter.

[89] Amrani B., Hassan F.E.H., Akbarzadeh H. (2007). First-principles investigations of the ground-state and excited-state properties of BeO polymorphs. J. Phys. Condens. Matter.

[90] Cai Y., Wu S., Xu R., Yu J. (2006). Pressure-induced phase transition and its atomistic mechanism in BeO: A theoretical calculation. Phys. Rev. B.

[91] Park C.J., Lee S.G., Ko Y.J., Chang K.J. (1999). Theoretical study of the structural phase transformation of BeO under pressure. Phys. Rev. B.

[92] Van Camp P.E., Van Doren V.E. (1996). Ground-state properties and structural phase transformation of beryllium oxide. J. Phys. Condens. Matter.

[93] Boettger J.C., Wills J.M. (1996). Theoretical structural phase stability of BeO to 1 TPa. Phys. Rev. B.

[94] Talwar D.N., Becla P. (2025). Systematic Simulations of Structural Stability, Phonon Dispersions, and Thermal Expansion in Zinc-Blende ZnO. Nanomaterials.

[95] Talwar D.N., Becla P. (2025). Composition Dependent Structural, Phonon, and Thermodynamical Characteristics of Zinc-Blende BeZnO. Materials.

[96] Kourouklis G.A., Sood A.K., Hochheimer H.D., Jayaraman A. (1986). High-pressure Raman study of the optic-phonon modes in BeO. Phys. Rev. B.

[97] Karch K., Bechstedt F. (1997). Ab initio lattice dynamics of BN and AlN: Covalent versus ionic forces. Phys. Rev. B.

[98] Pavone P., Karch K., Schiitt O., Windl W., Strauch D., Giannozzi P., Baroni S. (1993). Ab initio lattice dynamics of diamond. Phys. Rev. B.

[99] Erba A. (2014). On combining temperature and pressure effects on structural properties of crystals with standard ab initio techniques. J. Chem. Phys..

[100] Hao Y.-J., Chen X.-R., Cu H.-L., Bai Y.-L. (2006). First-principles calculations of elastic constants of c-BN. Phys. B.

[101] Wang Y.H., Xu H., Wang X.C., Jiang C.Z. (2009). High-pressure lattice dynamics and thermodynamic properties of zinc-blende BN from first-principles calculation. Phys. Lett. A.

[102] Weinstein B.A., Zallen R., Cardona M., Guntherodt G. (1984). Pressure-Raman Effects in Covalent and Molecular Solids, in Light Scattering in Solids IV.

